# Open-label, clinical phase I studies of tasquinimod in patients with castration-resistant prostate cancer

**DOI:** 10.1038/sj.bjc.6605322

**Published:** 2009-09-15

**Authors:** O Bratt, M Häggman, G Ahlgren, Ö Nordle, A Björk, J-E Damber

**Affiliations:** 1 Department of Urology, Helsingborg Hospital, Lund University, SE-25187 Helsingborg, Sweden; 2 Department of Urology, Uppsala University Hospital, SE-75185 Uppsala, Sweden; 3 Department of Urology, Malmö University Hospital, UMAS, SE-20502 Malmö, Sweden; 4 Active Biotech Research AB, SE-22007 Lund, Sweden; 5 Department of Urology, Bruna Stråket 11, Institute of Clinical Sciences, The Sahlgrenska Academy at University of Gothenburg, SE-41345 Gothenburg, Sweden

**Keywords:** prostate cancer, tasquinimod, anti-angiogenesis, phase I study

## Abstract

**Background:**

Tasquinimod is a quinoline-3-carboxamide derivative with anti-angiogenic activity. Two open-label phase I clinical trials in patients were conducted to evaluate the safety and tolerability of tasquinimod, with additional pharmacokinetic and efficacy assessments.

**Methods:**

Patients with castration-resistant prostate cancer with no previous chemotherapy were enrolled in this study. The patients received tasquinimod up to 1 year either at fixed doses of 0.5 or 1.0 mg per day or at an initial dose of 0.25 mg per day that escalated to 1.0 mg per day.

**Results:**

A total of 32 patients were enrolled; 21 patients were maintained for ⩾4 months. The maximum tolerated dose was determined to be 0.5 mg per day; but when using stepwise intra-patient dose escalation, a dose of 1.0 mg per day was well tolerated. The dose-limiting toxicity was sinus tachycardia and asymptomatic elevation in amylase. Common treatment-emergent adverse events included transient laboratory abnormalities, anaemia, nausea, fatigue, myalgia and pain. A serum prostate-specific antigen (PSA) decline of ⩾50% was noted in two patients. The median time to PSA progression (>25%) was 19 weeks. Only 3 out of 15 patients (median time on study: 34 weeks) developed new bone lesions.

**Conclusion:**

Long-term continuous oral administration of tasquinimod seems to be safe, and the overall efficacy results indicate that tasquinimod might delay disease progression.

In developed countries, prostate cancer is the second most frequently diagnosed cancer, and the third most common cause of death from cancer in men (Damber and Aus, 2008) with ∼240 000 new cases and ∼85 000 deaths a year from this cancer in Europe (Boyle and Ferlay, 2005). Metastatic disease is initially treated with androgen deprivation, which results in significant palliation for most patients (Huggins and Hodges, 1941; Denmeade and Isaacs, 2002). However, despite androgen deprivation and secondary hormonal manipulations, most patients eventually relapse on androgen ablative therapy and develop castration-resistant prostate cancer (CRPC). The median survival for patients with metastatic CRPC is 12–16 months from the time of diagnosis to death (Heidenreich and Schrader, 2003). No curative treatments are available at this stage of the disease. To date, docetaxel-based regimens have shown a modest survival advantage (Petrylak *et al*, 2004; Tannock *et al*, 2004). A lack of cancer cell specificity limits both dose and total length of treatment with cytotoxic agents. Thus, there is a high medical need for novel and well-tolerated agents that delay disease progression.

After neovascularisation was identified as a prerequisite for growth and metastasis of solid tumours (Folkman, 1972), direct or indirect inhibition of angiogenesis has become a viable therapeutic approach in anti-cancer therapy. By the inhibition of tumour neovascularisation, tumour growth is suppressed. There is a significant correlation between the density of microvessels and the incidence of metastases in prostate cancer (Weidner *et al*, 1993). These observations make the angiogenic pathway a rationale target for directing therapies in prostate cancer and an attractive approach to new treatment options (Figg *et al*, 2002; Aragon-Ching and Dahut, 2008).

Tasquinimod (ABR-215050; CAS number: 254964-60-8) is a quinoline-3-carboxamide derivative inhibiting tumour angiogenesis (Isaacs *et al*, 2006; Dalrymple *et al*, 2007). Although the anti-angiogenic mechanism of action of tasquinimod is presently unknown, it is not a vascular endothelial growth factor receptor (VEGFR) inhibitor. Pre-clinically, tasquinimod produced a robust inhibition of prostate cancer growth *in vivo* in a range of phenotypes and genotypes most characteristic of clinical tumours from patients with localised or metastatic, androgen receptor (AR)-positive or AR-negative, AR wild-type or mutant, as well as prostate-specific antigen (PSA)-positive or PSA-negative cancers. Treatment with a total daily dose of 1 mg kg^−1^ of tasquinimod over a period of 1 month decreased the tumour volume by at least 50% (Isaacs *et al*, 2006). This decrease in tumour volume was associated with a significant decrease in tumour blood vessel density. Similarly, the studies documented that a combination of androgen ablation or docetaxel with tasquinimod produced enhanced tumour growth retardation above that induced by either agent alone (Dalrymple *et al*, 2007).

In this study, we report the results of phase I studies in men with CRPC and PSA recurrence. The trials were conducted to define the safety and tolerability of tasquinimod when administered once daily and to serve as a basis for dose selection in future phase II studies. In addition, the studies investigated pharmacokinetics and the preliminary efficacy of tasquinimod.

## Patients and methods

### Patients

A total of 32 patients were enrolled for two multi-centre studies between February 2005 and January 2007. Eligibility criteria included informed consent, histologically or cytologically proven CRPC with PSA progression that was documented by at least two consecutive increases in PSA separated by at least 1 week during a maximum of 12 months before the initiation of therapy and a castrate level of testosterone (<50 ng per 100 ml) achieved by androgen-deprivation therapy through either surgical or pharmacological castration. Patients on a LHRH agonist were to continue androgen suppression during the studies. Patients who had received previous anti-androgen therapy were to have documented a subsequent increase in PSA after ceasing the anti-androgen. Patients were allowed on study without a detectable metastatic disease as assessed by bone scan or computed tomography (CT), provided they had documented increases in PSA. Patients had to have a score of 0 or 1 on the ECOG (Eastern Cooperative Oncology Group) performance status, and to have adequate bone marrow, renal and hepatic functions. Patients were excluded if they had clinical symptoms associated with prostate tumour progression, had received previous anti-cancer therapy (except for hormonal treatments), required systemic corticosteroids or warfarin, had previous significant cardiac events or had uncontrolled inter-current illness. The use of strong CYP3A4 inhibitors or inducers was not permitted while on study because of potential drug interaction. The study was conducted in accordance with the Declaration of Helsinki, Good Clinical Practice Guidelines, ethical committee approval and applicable local laws and regulations.

### Study design

**Figure 1:**
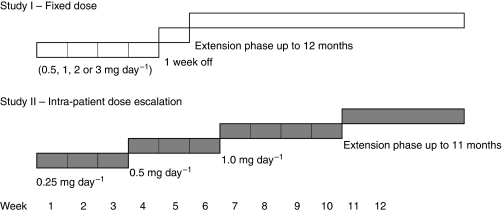
Study design of study I and study II.

The first of the two open-label studies conducted, study I, was a dose-escalation study to determine the maximum tolerated dose (MTD) of tasquinimod defined as the highest dose level at which no more than 1 out of 6 patients experienced a dose-limiting toxicity (DLT). Any drug-related grade 3 or 4 toxicity was considered a DLT. A cohort of six eligible patients was to receive an initial dose of 0.5 mg orally once daily. If the patients tolerated this dose level, next cohorts, each comprising six patients, were to receive escalating doses (1.0, 2.0 and 3.0 mg per day) of tasquinimod until the MTD was determined. The patients were to receive treatment with tasquinimod for 28 days, followed by a 1-week off period, and during an extension study thereafter for up to 12 months (Figure 1). Dose escalation would stop if 2 DLTs were observed within a cohort, and the preceding cohort would be expanded with an additional 10 patients to allow greater precision for the estimates of toxicity.

The second study conducted, study II, was an intra-patient stepwise dose-escalation study in eight patients. The patients were to receive an initial dose of 0.25 mg orally once daily for 3 weeks, and in the absence of treatment-related toxicity 0.5 mg per day for another 3 weeks and finally 1.0 mg per day for an additional 4 weeks. In the absence of treatment-related toxicity, patients were offered to continue their treatment at 1.0 mg per day during an extension study for another 11 months.

According to study protocols, treatment was discontinued because of disease progression (investigator's decision), treatment-related toxicity or withdrawal of consent.

### Procedures

In study I, patients were examined on days 1, 5, 8, 15, 23, 28, 35, 49, 63 and every 4 weeks thereafter until the final visit at 13 months. Safety evaluation was conducted at baseline and at each visit thereafter; that is, all patients underwent a physical examination, and vital signs, haematology, clinical chemistry, coagulation, urinalysis and ECG were evaluated. In addition, serum PSA values were measured at days 1, 15, 28, 35 and at every 4 weeks thereafter until the final visit. A PSA response was defined as a decrease in PSA >50% from the baseline value and was confirmed by a second PSA value 4 weeks later (Bubley *et al*, 1999). The time to PSA progression was defined as the time from randomisation to a ⩾25% increase in serum PSA from baseline or from on-treatment nadir PSA value (Scher *et al*, 2008). The PSA doubling time (PSADT) was calculated using the log-slope method (Daskivich *et al*, 2006). Regression analyses were conducted for the historic and on-study PSA data. Historic regression analysis was conducted using weighted linear regression. More weight was given to samples, which were closer to the screening visit. The weights were set at 1, 0.5 and 0.25 for values not older than weeks 16, 32 and 52, respectively, before the first dose. Values older than week 52 before screening were excluded. In the on-study regression analysis, the same weight (weight=1) was given to all PSA values collected throughout the study.

Radiological imaging with bone scan was performed at screening and at every 2 months until the final visit. The overall time to PSA progression and radiographic progression was defined using Kaplan–Meier methods.

In study II, patients visited the study site on days 1, 5, 8, 15, 26, 29, 36, 47, 50, 57, 71 and at 3 months and every 2 months thereafter until the final visit at 12 months. The procedure for safety evaluation was the same as in study I. PSA measurements were performed at baseline, on day 71 and every 2 months thereafter.

In study I, extensive pharmacokinetic sampling was performed on days 1 and 28. Besides, a blood sample (pre-dose) was collected at all visits. Tasquinimod concentrations in plasma samples were determined using a validated liquid chromatography mass spectrometry/mass spectrometry method. Briefly, the plasma sample was prepared for injection onto the column by protein precipitation, followed by centrifugation. Quantitation was based on stable isotope dilution. The workable concentration range for the method was 1–2500 nmol l^−1^ with inter-batch precision and accuracy in the same concentration interval being 6.1 and 102.9%, respectively. Pharmacokinetic parameters, including area under the concentration–time curve (AUC), maximum concentration (C_max_), time to C_max_ (*t*_max_) and elimination half-life (*t*_1/2_) were evaluated by non-compartmental analysis using WinNonlin professional software version 5.0.1 (Pharsight Corp., Mountain View, CA, USA).

### Statistical evaluations

All variables are presented using descriptive statistics. No formal statistical tests were conducted in these studies.

## Results

### Patient characteristics

In total, 32 patients were enrolled for these studies. Baseline clinical and biological characteristics are described in Table 1. Patients ranged in age from 46 to 82 years, and all of them were Caucasian. There were no clinically meaningful differences between the patients in baseline characteristics in the two studies, including factors with established prognostic importance in prostate cancer (Smaletz *et al*, 2002; Armstrong *et al*, 2007). The median PSA concentration at study entry was 19 ng ml^−1^ (range: 2.6–1400). Pre-treatment median PSADT was 25 weeks (range: 8 to >999). Two patients had a decline in PSA just before entering the study, in one patient after discontinuing anti-androgen treatment. Bone scan was performed in study I, and 50% of the patients had bone metastatic disease at baseline.

### Efficacy

**Figure 2:**
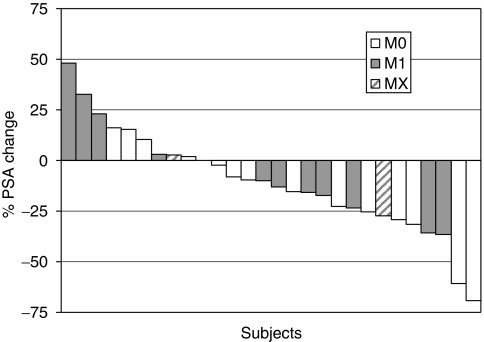
Waterfall plot of best PSA response per subject, with each bar representing one patient's lowest percentage decline after treatment initiation (*n*=28). The 28 patients are divided into three groups (M0, M1, and MX) according to distant metastasis on bone scan at baseline.

**Figure 3:**
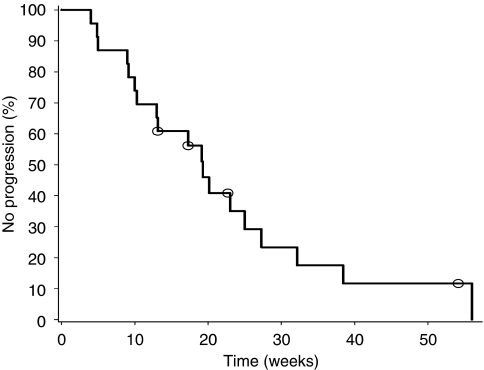
Kaplan–Meier graph of time to PSA progression (PSA increase >25% over baseline or on-treatment NADIR) in patients entering the extension phase (*n*=23; censored subjects=circle). The median PSA PFS was 19 weeks.

Two patients in the 0.5 mg dose group in study I had a PSA decline of >50% at 10 and 26 weeks, lasting for >7 and 13 weeks, respectively. An additional patient had a decline of >30% at 9 weeks lasting for 12 weeks. The best PSA response in all patients treated for >15 days (*n*=28) are graphically shown in the waterfall chart in Figure 2. The estimated proportion of patients without PSA progression at week 18 was 56%. The median PSA progression-free time was 19 weeks (95% CI, 13–25 weeks; *n*=23) in patients entering the extension phase (Figure 3). The median pre-treatment PSADT was 25 weeks, and the median PSADT at 18 weeks was 65 weeks. In all, 18 patients had prolonged PSADT while on study; in 5 patients PSADT was shortened.

**Figure 4:**
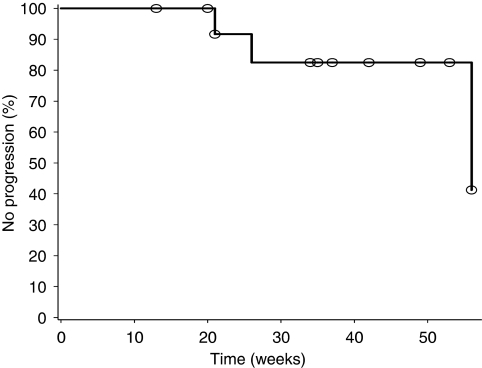
Kaplan–Meier graph of time to new bone lesions in patients treated at a fixed dose of 0.5 mg per day (*n*=15; censored subjects=circle).

Bone scans were performed at baseline and thereafter at every 2 months in the fixed 0.5 mg dose group. In all, 7 (47%) out of 15 patients continuing during the extension study had positive scans at baseline. It is noteworthy that there was no finding of bone scan progression at the end of study in 12 (80%) patients (median treatment time: 34 weeks; range: 15–54) (Figure 4). One (14%) patient with a positive scan at baseline developed new lesions at week 53; two (25%) patients with negative scans at baseline developed new bone lesions at weeks 13 and 21, respectively. No patient in the trial developed tumour-related pain requiring treatment with opioid drugs. The median time to PSA progression for patients with and without bone lesions at baseline was 9 (95% CI, 5–23 weeks; *n*=7) and 23 weeks (95% CI, 13–56 weeks; *n*=8), respectively. All three patients who developed new bone lesions also progressed by PSA criteria.

Notably, patients treated with tasquinimod responded with a decrease in serum lactate dehydrogenase (LDH) levels. Compared with baseline, patients had a LDH mean (±s.e.m.) decrease of 20±3% at 4 months.

### Maximum tolerated dose

In study I, 24 patients received tasquinimod at fixed doses of 0.5 (17 patients) or 1.0 mg (7 patients) orally once daily on a continuous dosing schedule for 28 days. Patients treated at the 0.5 mg dose level tolerated the therapy without DLT. In the cohort comprising seven patients at a dose level of 1.0 mg per day, two patients experienced DLT as pre-defined by protocol (Table 2). One patient had an asymptomatic CTC grade 3 elevation in amylase on day 23. He recovered without any clinical intervention within 2 weeks. The second DLT patient had chest and back pain and was hospitalised on day 10 because of a grade 3 cardiac arrhythmia. The patient had periods of supraventricular tachycardia. There were no signs consistent with myocardial infarction. He received treatment with *β*-blockers and recovered 2 weeks later. Owing to these events, the remaining patients in the 1.0 mg dose group were discontinued prematurely. The MTD of tasquinimod was determined to be 0.5 mg once daily.

The 0.5 mg dose group was expanded with an additional 11 patients. All patients in the 0.5 mg dose group completed the 28-day treatment period. After 1-week of treatment, patients without signs of tumour progression were offered to continue tasquinimod treatment for up to 12 months. In all, 15 (88%) out of the 17 patients entered the extension phase. The mean number of days with intake of study drug in the 0.5 and 1.0 mg dose groups was 240 and 11, respectively. Three (20%) patients completed the 12-month extension study.

### Safety results

Inflammation (41%) was the most common adverse event associated with tasquinimod in the fixed 0.5 mg dose group during the first 4 weeks of treatment (Table 3). In this study, inflammation is defined as increased levels of laboratory markers, constituting laboratory changes above the upper limit normal not associated with clinical symptoms requiring any clinical intervention. In the fixed 1.0 mg dose group, nausea (57%) and fatigue (43%) were the most common adverse events. The majority of these events was CTC grade 1 in severity, transient and occurred early after the start of treatment.

The most commonly occurring adverse events assessed as related to tasquinimod in the intra-patient dose-escalation study (study II) were myalgia (25%), hypoesthesia (25%) and pain in extremity (25%).

Compared with baseline, patients in both study I and study II had mean haemoglobin decreases of 12 and 16 g l^−1^, respectively. These changes occurred within 2 weeks of the initiation of treatment and were stabilised 6 weeks later, except for one case of anaemia for which a blood transfusion was required.

In the fixed 0.5 mg dose group, there were decreases in mean systolic and diastolic blood pressure levels starting during the first weeks of treatment continuing throughout the study. At 3 months, the mean (±s.e.m.) systolic blood pressure was decreased by 20.6±5.3 mmHg and the diastolic blood pressure by 5.3±2.4 mmHg. At 10 weeks in the intra-patient dose-escalation study, there was also a similar decrease in mean (±s.e.m.) systolic and diastolic blood pressure levels, that is, 18.1±6.4 and 7.4±2.0 mmHg, respectively. No other changes in vital signs (mean pulse, mean oral body temperature, mean body weight) or ECG parameters were considered clinically relevant.

In study I, five patients experiencing severe adverse events (CTC grade 3) were documented (Table 3). No adverse event of CTC grade 4 was observed. Two patients in the 0.5 mg dose group developed asymptomatic CTC grade 3 elevations in amylase on day 8. These abnormalities resolved without treatment interruption and were normalised within 7 days. A third patient experienced back pain. He recovered with sequelae in one day. The two patients who experienced DLT are described above.

In study II, three SAEs were recorded in two patients during the study in which a causal relationship with tasquinimod could not be excluded. One patient experienced two SAEs, namely musculoskeletal pain (CTC grade 2) and chest pain (CTC grade 3). The patient discontinued the study because of the pain and recovered. Another patient experienced an SAE of cerebral infarction (CTC grade 3). Approximately 1 year after starting to take the study drug, this patient noted aphasia and poor coordination of his left body half and was hospitalised. A CT scan of the brain showed no pathological changes. He received treatment with enalapril, acetylsalicylic acid and simvastain. The patient recovered and was discharged from the hospital 3 days later.

### Plasma PK

In the 0.5 mg fixed dose group, the maximum plasma concentration (C_max_) (mean: 0.26 *μ*mol l^−1^, *n*=17), was attained at 2.6 h. The oral clearance (CL/F) was 0.28 l h^−1^ (mean) and the oral volume of distribution (V/F) was 15 l (mean), resulting in an elimination half-life of 40±16 h (mean±s.d.). The AUC at steady state amounted to 4.8 *μ*mol h l^−1^ (mean). The accumulation ratio in plasma level (AUC_24 h_) by multiple dosing was 2.8 and was expected on the basis of the half-life of tasquinimod. The steady-state plasma concentrations of tasquinimod showed no time dependency in the exposure during up to 1 year of treatment: the state pre-dose (trough) concentration of tasquinimod (mean: 0.19 *μ*mol l^−1^) determined during 12 months of daily administration was the same as after 14 days of administration. The pharmacokinetic properties were not affected by food intake. The pre-dose plasma concentrations of tasquinimod determined after 14 days of daily administration at 0.25, 0.5 and 1.0 mg in the intra-patient dose-escalation study were 0.13±0.02, 0.23±0.03 and 0.42±0.05 *μ*mol l^−1^ (mean±s.e.m., *n*=8), respectively. No essential dose dependency of the pharmacokinetics was observed.

## Discussion

Structurally, tasquinimod is related to roquinimex (linomide), which was tested in a series of phase II and III trials in patients with advanced renal cell carcinoma (de Wit *et al*, 1997; Pawinski *et al*, 1997) and in those with multiple sclerosis (MS) (Noseworthy *et al*, 2000). In patients with renal cell cancer, the efficacy was modest with influenza-like symptoms of myalgia, arthralgia and fatigue being the most common adverse events. Roquinimex was terminated because of unanticipated serious cardiopulmonary toxicities detected in studies on MS patients. The mechanism of these adverse effects is unknown, but may be related to a proinflammatory reaction seen in a polyarthritis model mimicking proinflammatory side effects in the dog. Doses of roquinimex that inhibit tumour growth in the mouse induce a proinflammatory reaction in the dog. Its main characteristics are fever, neutrophilia and an increase in acute-phase reactant levels (Jönsson *et al*, 2004). When the toxicity of tasquinimod was assessed in dogs, only occasional increases in acute-phase reactants and white blood cell counts were observed (unpublished observation), and hence inducing systemic inflammatory reactions seems to have less significance for tasquinimod. Pre-clinically, tasquinimod is 30–60 times more potent than roquinimex in its anti-tumour efficacy (Isaacs *et al*, 2006). Thus, compared with roquinimex, significant improvements in both anti-angiogenic and anti-prostate cancer activities and safety aspects have been accomplished in tasquinimod.

On the basis of the effects of tasquinimod against human prostate cancer xenografts, it has entered clinical development as therapy for prostate cancer. These phase I trials were conducted to evaluate the safety and preliminary efficacy of tasquinimod in men with asymptomatic CRPC without previous systemic chemotherapy.

In these phase I studies, tasquinimod could be safely administered at 0.5–1.0 mg per day during long-term treatment. Most adverse events occurred within the first 4 weeks of treatment. Of a total of 71 related adverse events in the fixed 0.5 mg dose group, 76% were recorded until day 35. Raised inflammatory markers not associated with clinical symptoms are the most frequent adverse effects, whereas fatigue, myalgia and nausea are the most common symptomatic adverse events. Nausea seemed to be related to taking tasquinimod under fasting conditions.

On the basis of the observations that most laboratory changes were transient, not associated with clinical symptoms and occurred early after the start of treatment, an intra-patient stepwise dose-escalation study was conducted to determine whether these laboratory changes could be reduced. Given the small sample size in the intra-patient dose-escalation group, it is not possible to provide a precise estimate, but the intra-patient dose-escalation regimen seemed to decrease the number of related adverse events per patient per month. Thus, the intra-patient dose-escalation regimen may prove beneficial to future trials with tasquinimod.

Several patients developed asymptomatic elevations in amylase ∼1 week after the start of treatment. Amylase levels normalised without dose interruption within a couple of weeks. The reasons for this transient elevation in amylase are unknown. As hyperamylasaemia was not associated with clinical symptoms and no patient developed acute pancreatitis, it is possible that the asymptomatic increase in amylase should not be considered as a relevant DLT. Notably, asymptomatic increases in serum levels of amylase and lipase have been observed with the anti-angiogenic agents such as sorafenib, sunitinib, and temsirolimus and may represent a class effect (Escudier *et al*, 2006; Bhojani *et al*, 2008). However, other side effects noted with VEGFR inhibitors and agents targeting VEGF such as skin rashes, hand–foot skin reactions, diarrhoea, hypertension and neuropathic changes (Kane *et al*, 2006; Rock *et al*, 2007) were not observed with tasquinimod. Thus, there seems to be a substantial difference in the side effect profile of tasquinimod compared with VEGFR inhibitors.

Several anti-angiogenic therapies are currently under intense clinical investigation both as stand-alone therapies and as adjuvants to chemotherapy to enhance the anti-tumour activity of the latter. In general, the PSA response of the anti-angiogenic agents as stand-alone therapies in metastatic androgen-independent prostate cancer is modest (Figg *et al*, 2001; Reese *et al*, 2001; Steinbild *et al*, 2007; Dahut *et al*, 2008).

The present trials were primarily designed to evaluate the safety of tasquinimod. However, efficacy was addressed by measuring PSA levels in all patients and by bone scan in patients treated at 0.5 mg per day. Preclinical data suggest that the effect of tasquinimod on serum PSA levels is related to its inhibitory effect on tumour growth (Dalrymple *et al*, 2007), which supports a hypothesis that a clinical change in PSA slope or PSADT is indicative of a therapeutic response to tasquinimod. In the current studies, 56% of patients were progression free by PSA criteria (<25% increase) at 18 weeks. The median PSADT up to 18 weeks was 65 weeks compared with the median pre-treatment PSADT of 25 weeks. In this respect, tasquinimod as a stand-alone therapy seems to be superior to most other anti-angiogenic agents both in terms of PSA progression-free survival and PSA declines. Repeated radiological imaging with bone scan was performed in patients who received the fixed dose of 0.5 mg per day. Seven of these patients had a positive bone scan at baseline, whereas eight patients had negative scans. Only three (20%) patients had radiographic progression on bone scans. Two of these patients had negative bone scans at baseline. No patient had evidence of tumour-related pain. However, this study did not conform to the recent recommendation of the PCWG2 (Prostate Cancer Clinical Trials Working Group) to assess the progression on bone scan (Scher *et al*, 2008). Therefore, it is important that future studies be harmonised with these novel recommendations. Two patients had PSA progression (at 5 and 9 weeks, respectively) before radiographic progression (at 53 and 13 weeks, respectively), and one patient had PSA and radiographic progression at the same time (at 21 weeks). Two of these patients had negative bone scans at baseline. No patient had evidence of tumour-related pain.

Lactate dehydrogenase levels may be a marker for tumour burden (Smaletz *et al*, 2002; George *et al*, 2005). It was observed that patients experienced a decrease in their LDH levels during treatment. Interestingly, LDH levels are not affected in healthy subjects treated with tasquinimod (data not shown). Alkaline phosphatase levels were only affected to a minor extent.

In conclusion, there is a need for novel therapies for treating metastatic prostate cancer. Anti-angiogenic therapy has demonstrated its potential as a therapy to stabilise or delay disease progression. Although caution must be exercised in interpreting early data, treatment in the form of long-term continuous oral administration of tasquinimod seems to be safe and well tolerated with a favourable pharmacokinetic profile, and overall efficacy results indicate that tasquinimod might delay disease progression. On the basis of the encouraging phase I results, a randomised phase II program is currently being performed to further determine the efficacy and safety of tasquinimod in asymptomatic patients with metastatic CRPC. The tasquinimod profile demonstrated may also prove beneficial to future trials combining tasquinimod with chemotherapy, radiation, anti-hormonal or other anti-angiogenic agents.

## Conflict of interest

The authors declare no conflict of interest.

## Figures and Tables

**Table 1:** Baseline characteristics

	**Study I^a^ (*n*=24)**		
**Variable**	**0.5 mg per day (*n*=17)**	**1.0 mg per day (*n*=7)**	**Study II^b^ (*n*=8)**
*Age, years*			
Median	72	77	73
Range	46–82	58–82	61–78
			
*PSA, ng per 100 ml*			
Median	19	112	9.4
Range	2.6–1400	6.3–1188	5.0–25
			
*PSADT, weeks*			
Median	23	27	29
Range	8 to >999	13–81	9 to >999
			
*Gleason's score*			
Median	7	7	7
Range	6–9	6–10	6–9
			
*Gleason's score, n*			
⩽6	1 (6%)	1 (14%)	1 (13%)
7	6 (35%)	2 (29%)	3 (38%)
8–10	6 (35%)	2 (29%)	2 (25%)
Indeterminate	4 (24%)	2 (29%)	2 (25%)
			
*Bone metastases* c			
M0	8 (47%)	4 (57%)	3 (38%)
M1	9 (53%)	3 (43%)	3 (38%)
MX	0	0	2 (25%)
			
*ECOG, n*			
0	17 (100%)	6 (86%)	7 (88%)
1	0	1 (14%)	0
Indeterminate	0	0	1 (13%)
			
*Haemoglobin, g l* ^*−1*^			
Median	142	127	133
Range	124–154	107–140	126–148
			
*Alkaline phosphatase,* μ*kat l*^*−1*^			
Median	1.2	1.5	1.6
Range	0.8–10	0.7–3.2	0.9–2.1
			
*Lactate dehydrogenase,* μ*kat l*^*−1*^			
Median	3.5	3.7	2.9
Range	2.1–5.7	3.1–4.3	2.2–3.5

Abbreviations: ECOG=Eastern Cooperative Oncology Group; PSA=prostate-specific antigen; PSADT=prostate-specific antigen doubling time.

a Fixed dose groups.

b Intra-patient dose-escalation group.

c No soft tissue lesions were observed.

**Table 2:** No. of patients with tasquinimod related CTC grade 3 adverse events/SAE/DLT

	**Dose level mg per day**	**No. of patients**	**SAE**	**DLT**
*Study I*				
Back pain	0.5	1		
Hyperamylasaemia	0.5	2		
	1.0	1		1
Sinus tachycardia	1.0	1	1	1
				
*Study II*				
Cerebral infarction	1.0	1	1	
Chest pain	1.0	1	1^a^	

Abbreviation: DLT=dose-limiting toxicity.

a This patient also experienced another Serious Adverse Event (SAE), musculoskeletal pain Common Toxicity Criteria (CTC) grade 2.

**Table 3:** Percentage of patients with tasquinimod-related adverse events (only symptoms occurring in two or more patients (*n*=32))

		**Fixed 0.5 mg per day (*n*=17)**		**Fixed 1.0 mg per day (*n*=7)**		**Fixed 0.5 mg per day extension (*n*=15)**		**0.25–1.0 mg per day (*n*=8)**	
**SOC**	**Dictionary-derived term**	**Grades 1 and 2 (%)**	**Grade 3 (%)**	**Grades 1 and 2 (%)**	**Grade 3 (%)**	**Grades 1 and 2 (%)**	**Grade 3 (%)**	**Grades 1 and 2 (%)**	**Grade 3 (%)**
Blood and lymphatic system disorders	Anaemia	18		29		33			
Gastrointestinal disorders	Abdominal pain	12							
	Constipation	12				7		13	
	Nausea	6		57				13	
General disorders and administration site conditions	Fatigue	18		43		7		13	
	Feeling cold	6		29					
	Inflammation	41		29		33			
	Pain	12							
	Pyrexia	6						13	
Investigations	Blood fibrinogen increased	24				7			
	CRP increased	24							
	Hb decreased	6				7			
	Monocyte count increased	12				7			
	Neutrophil count increased	12				7			
	Platelet count increased	6				13			
	ESR increased	18				7			
	WBC count increased	24				7			
Metabolism and nutrition disorders	Hyperamylasaemia		12		14	7			
	Hypercalcaemia	6		14					
Musculoskeletal and connective tissue disorders	Arthralgia			14				13	
	Muscular weakness	6				7			
	Musculoskeletal stiffness	6		14					
	Myalgia	18				7		25	
	Pain in extremity	6						25	
Nervous system disorders	Headache	6				7		13	
	Hypoesthesia							25	
Psychiatric disorders	Insomnia	6				7			
Skin and subcutaneous tissue disorders	Eczema	6				7			

Abbreviations: Hb=haemoglobin; WBC=white blood cell; CRP=C-reactive protein; ESR=erythrocyte sedimentation rate; SOC=system organ class.
